# Modeling an enhanced ridesharing system with meet points and time windows

**DOI:** 10.1371/journal.pone.0195927

**Published:** 2018-05-01

**Authors:** Xin Li, Sangen Hu, Wenbo Fan, Kai Deng

**Affiliations:** 1 Department of Electronic Engineering, The Hong Kong Polytechnic University, Hong Kong, China; 2 School of Civil and Transportation Engineering, Guangdong University of Technology, Guangzhou, China; 3 School of Transportation and Logistics, Southwest Jiaotong University, Chengdu, China; 4 School of the Gifted Young, University of Science and Technology of China, Hefei, China; Beihang University, CHINA

## Abstract

With the rising of e-hailing services in urban areas, ride sharing is becoming a common mode of transportation. This paper presents a mathematical model to design an enhanced ridesharing system with meet points and users’ preferable time windows. The introduction of meet points allows ridesharing operators to trade off the benefits of saving en-route delays and the cost of additional walking for some passengers to be collectively picked up or dropped off. This extension to the traditional door-to-door ridesharing problem brings more operation flexibility in urban areas (where potential requests may be densely distributed in neighborhood), and thus could achieve better system performance in terms of reducing the total travel time and increasing the served passengers. We design and implement a Tabu-based meta-heuristic algorithm to solve the proposed mixed integer linear program (MILP). To evaluate the validation and effectiveness of the proposed model and solution algorithm, several scenarios are designed and also resolved to optimality by CPLEX. Results demonstrate that (i) detailed route plan associated with passenger assignment to meet points can be obtained with en-route delay savings; (ii) as compared to CPLEX, the meta-heuristic algorithm bears the advantage of higher computation efficiency and produces good quality solutions with 8%~15% difference from the global optima; and (iii) introducing meet points to ridesharing system saves the total travel time by 2.7%-3.8% for small-scale ridesharing systems. More benefits are expected for ridesharing systems with large size of fleet. This study provides a new tool to efficiently operate the ridesharing system, particularly when the ride sharing vehicles are in short supply during peak hours. Traffic congestion mitigation will also be expected.

## 1. Introduction

### 1.1 Background

The study on ridesharing has received much attention since the rise of e-hailing services (e.g., Uber, Lyft, and DiDi). Using ubiquitous real-time communications, private car drivers and ridesharing service seekers are being automatically matched according to their itineraries and schedules. The ridesharing system is recognized to possess many advantages to participants (both drivers and passengers), to society, and to the environment in terms of saving travel cost, reducing travel time, mitigating traffic congestions, conserving fuel, and reducing air pollution.

In this paper, we focus on an enhanced ridesharing system that respect (i) users preferable time windows and (ii) meet points where passengers are willing to walk to/from for collective pickup or drop-off. The consideration of the above two constraints is necessary and important to furnish a more user-friendly and efficient ridesharing service than the conventional modes. For instance, a preferred departure time window is very commonly associated with commuters (or travelers on business trip) at their origins, from which they won’t be able to be picked up too early on one hand; and on the other hand, they also will refuse to wait too long before served. In addition, a latest arrival time is often specified at the destinations of ridesharing participants. As for including the meet point into the proposed ridesharing system, it is expected to take advantage of constructing routes with smaller detours, while maintaining a satisfactory range of walking for riders. From the theoretical prospective, the conventional door-to-door ridesharing system becomes a special case of the proposed system when each passenger is assigned to his/her own origin/destination as the meet point. The proposed ridesharing problem seeks to determine the optimal route for each vehicle and the optimal assignment of passengers to vehicles as well as to the meet points.

### 1.2 Literature review

This section reviews the existing literature that is relevant to our work. The ridesharing problem can be mathematically modeled by one of the well-known optimization problems: the vehicle routing problem with pickup and delivery with time windows (VRPPDTW) or simply, pickup and delivery problem with time windows (PDPTW) [1]. The PDPTW has a wide range of applications in the fields of transportation and logistics (see the recent review by Mahmoudi and Zhou [2]). For instance, applications in cargo routing and scheduling include [3–6]; transportation of elderly or handicapped people [7–8]; and school bus routing and scheduling [9–10]. Specifically in the field of passenger transportation, the PDPTW could also be observed as the dial-a-ride (DAR) problem in the literature, which generally concerns designing transportation service for riding requests from specific pickup locations to specific drop-off locations, while imposing certain requirements (e.g., time window and vehicle capacity constraints)[11]. Ridesharing problem adds new constraints from the private driver side to the DARP, such as the maximum detour constraint from the driver’s own itinerary (which may not be a concern to commercial vehicles operated by a company in case of DARP). Other than that, ridesharing problem shares most of the properties with DARP.

DARP literature is extensive and has many variants, which can be classified based on several characteristics, e.g., single vehicle or multiple vehicles, static or dynamic demands, and homogeneous or heterogeneous vehicle fleet. Early studies were mainly about the single-vehicle DARP [12–14], which is a special case of the multiple vehicles DARP [15–16]. The most of cases propose discrete models and have been proved to be NP-hard [12]. For the static DARP (e.g., [17]), all riding demands are known in advance with specified time windows for pickup and/or delivery, while for the dynamic DARP (e.g., [11]), some requests are allowed to emerge or disappear progressively during the service process. Compared to the homogeneous DARP, heterogeneous DARP (H-DARP) [18] recognizes the heterogeneity of vehicles in terms of capacities, performances, and other various resources (e.g., wheelchair spaces). Additional considerations, such as routing properties [19] and stochastic properties [20–21], have also been studied in DARP literature to build models with more realistic characteristics (accounting for transfers among routes and travel time uncertainties, for instance).

When designing DAR systems (ridesharing as well), a commonly used objective is to minimize the operational costs (e.g., in terms of total travel distances) that are subject to full demand satisfaction and constraints [22]. Alternatively, a number of optimizations consider fleet efficiency, such as minimizing fleet size [23] or maximizing vehicle usage efficiency. In a dynamic context, the number of requests served in time may be maximized [24–25], or their total trip time may be minimized [26]. Extending from the DARP with a single objective (which is either cost-based or efficiency-based), many studies introduced multiple objectives to optimize the service quality as well. For instance, the most frequent quality-related objectives include minimizing total user waiting time (caused by deviations from their preference time), minimizing vehicle idle time [27], and maximizing the number of ride-matches. The multi-objective DARP is often formulated into a single weighted-sum objective function to facilitate the solving process.

Solution approaches to DARP can be basically divided into three classes: exact algorithms, heuristics, and meta-heuristics. Due to the NP-hardness of discrete-based DARP models, exact algorithms are promising only for the single vehicle problem or small-size multi-vehicle problems. For instance, Psaraftis [12–13] presented exact dynamic programming (DP) solution algorithm for a single-vehicle DARP to minimize a weighted sum of the total service time and the total waiting time for all customers. A column generation approach is developed by Dumas et al. [28] for a multi-vehicle DARP, of which the objective is to minimize the total travel cost. Cordeau [29] utilizes the branch-and-cut algorithm to solve a medium-size DARP with up to 48 requests. Later only, the branch and cut and price algorithm is further proposed with higher computation capability for the PDPTW [30].

For complex DARP with additional constraints, most research attentions are devoted to developing heuristics and meta-heuristics due to the computational efficiency constraint. For instance, Jaw et al. [31] develop a sequential insertion heuristics by sorting users according to their earliest pickup time and iteratively optimizing the routing plan. Kim and Haghani [32] and Wong et al. [33] apply the sequential insertion heuristics to a time-dependent DARP and a dynamic DARP, respectively. Luo and Schonfeld [34] establish a rolling horizon insertion strategy for dynamic DARP. Another type of heuristics is cluster-first-route-second heuristics, which split the DARP into separate phases: (i) generating clusters and (ii) solving the vehicle routing subproblem. Contributing works of this type include [35–37].

Heuristics are, however, easily be trapped in local optima. In light of this, meta-heuristic algorithms are more often developed nowadays by incorporating these classical heuristics in the solution framework (e.g. to generate an initial solution). A pioneering work is that by Cordeau and Laporte [38], who propose a Tabu Search (TS) algorithm for the DARP. Since then, a variety of meta-heuristics have been developed for contending with many DARP variants. Generally, they can be grouped into two categories: local-search based and population based algorithms. The former includes the Variable Neighborhood Search (VNS) algorithm, Large Neighborhood Search (LNS) algorithm, deterministic annealing, and evolutionary local search. The latter mainly consists of various enhanced genetic algorithms. Detailed reviews are referred to [22].

Previous research has made many important contributions to this challenging problem along with different formulation or solution approaches. The conventional ridesharing system, however, may not work well providing door-to-door service in dense-populated urban areas, where huge waste of time delays may be caused by frequent stops at very closely located pickup and drop-off points in neighborhood streets. Particularly, the door-to-door ridesharing system won’t be feasible in the environment of gated communities, where riders have to gather at some commonly recognized locations (e.g., main gates and major intersections, if there are no designated ones). Moreover, how to efficiently schedule the ride sharing services in the condition of running short of the available dispatching vehicles is also a challenge to both operators and users. Most of the existing works have ignored the location selection problem when neighborhood riders have to share pickup/drop-off points (namely meet points), and some or all of them are willing to walk to and from the meet points to facilitate the pickup and drop off process. Taking this arrangement into account is able to extend the traditional ridesharing system to a more flexible system providing services of door-to-door, door-to-meet-point, meet-point-to-door, and meet-point-to-meet-point. By trading off the walking cost and the benefit of pickup/drop-off saving, the meet-point ridesharing system is expected to minimize the total system cost, particularly in dense urban area with many closely located requests (in peak hours, for instance). It is also expected to better solve the problem of lacking of dispatching vehicles, and to maximize the limited resources.

The setting of meet points imposes new challenge to the modeling efforts in terms of location choice, time coordination, and passenger assignment. So far, very few studies can explicitly account for the meet point setting in ridesharing system optimization. An example is Stiglic et al. [39], who demonstrated the considerable benefits of introducing meet points in a ridesharing system. Our study even complicates the problem by further introducing preferable time windows for ride requests other than the traditional of earliest-pickup and latest-arrival time constraint as the treatment in [39]. This imposes new challenges in matching the riders and ridesharing vehicles as well as riders that can be assigned to the same meeting point within their overlapping time windows. Another example is Daganzo [40], who developed continuum models for a DAR with meet points (called checkpoints). Although system performance metrics (e.g., total travel time) were analytically derived, no routing plans and specific locations of meet points can be generated from these models.

### 1.3 Research motivation

In light of the above, this study aims to develop a new model to design the appropriate routing plans for the flexible ridesharing system. The contributions of our paper are as follows:

(i) The proposed model incorporates meet points in ridesharing system by allowing riders free of choosing to walk to the meet points or to be directly picked up/dropped off at their origins/destinations;
(ii) The model simultaneously considers riders’ desired pickup time windows and drop-off time windows as well as the maximum route travel time constraint from the driver side; and
(iii) A meta-heuristic algorithm is developed and evaluated in the application on a medium-size network.

The remainder of the paper is organized as follows. Section 2 first describes the ridesharing system in this study, and then formulates the optimization problem as a mixed integer linear program (MILP). Section 3 presents an enhanced Tabu Search (TS) algorithm embedded with another heuristic algorithm that is used to generate high-quality initial solutions. Section 4 summarizes the results of numerical experiments, in which the solution quality and computation efficiency of the proposed meta-heuristic algorithm are demonstrated. The merits of introducing meet points to ridesharing systems are also illustrated. Conclusions are drawn in the final section.

## 2. Problem statement

### 2.1 Description of the ridesharing system

Consider a complete network, *G* = (***V*,*A***)[41], where ***V*** is the set of vertices, *i*,*j*,*m*,*s* ∈ ***V***, and is partitioned as ***V*** = ***V***_***p***_ ∪ ***V***_***d***_ ∪ ***V***_***o***_ ∪ ***V***_***e***_, where ***V***_***p***_ is the subset of vertices associated with pick-up locations of seekers; ***V***_***d***_ is the subset of vertices associated with drop-off locations of onboard passengers; ***V***_*o*_ is the subset of vertices associated with the origin locations of drivers; ***V***_*e*_ is the subset of vertices associated with the destinations of drivers. Let *T* be the set of all ridesharing vehicles, *P* be the set of all demand announcements. Each seeker is associated with an earliest departure time window [$e_{p}^{pick},l_{p}^{pick}$] from the corresponding pickup location, and also a latest drop-off time $U_{p}^{drop}$ at their corresponding drop-off locations. A maximum trip duration *T*_*t*_ is specific to the driver of vehicle *t*.

### 2.2 Notation

Table 1 summarizes the notation used in this paper.

**Table 1:** Variables and parameters in the model.

*Indices*		
*i*,*j*,*m*.*s*		Vertex index
*p*		Passenger index
*t*		Vehicle index
*Sets*		
***P***		Set of passengers
***T***		Set of vehicles
***V***		Set of vertices, ***V*** = ***V***_*p*_ ∪ ***V***_*d*_ ∪ ***V***_*o*_ ∪ ***V***_*e*_
***V***_***d***_		Set of drop-off locations ***V***_*d*_ ⊂ ***V***
***V***_***e***_		Set of drivers’ destinations ***V***_*e*_ ⊂ ***V***
***V***_***o***_		Set of drivers’ origins ***V***_*o*_ ⊂ ***V***
***V***_***p***_		Set of pick-up locations ***V***_*p*_ ⊂ ***V***
*Parameters*		
*D*_*ij*_		Distance from vertex *i* to vertex *j*; *i*,*j* ∈ ***V***;
$e_{p}^{pick}$		The earliest pick-up time of passenger *p* at the corresponding pick-up location
$l_{p}^{pick}$		The latest pick-up time of passenger *p* at the corresponding pick-up location
*Q*_*t*_		Capacity of vehicle *t*; *t* ∈ *T*
*S*		Average walking speed;
*T*_*t*_		Maximum travel time of vehicle *t*; *t* ∈ *T*
*Tim*_*ij*_		Vehicular time from vertex *i* to vertex *j*; *i*,*j* ∈ ***V***;
$U_{p}^{drop}$		The latest drop-off time of passenger *p* at the corresponding destination
*Decision Variables*		
$d_{i}=\{\begin{array}{c} 1\\ 0 \end{array}$	If the pick-up and drop-off locations is selected as visited stop;	
	Otherwise;	
$r_{ij}^{p}=\{\begin{array}{c} 1\\ 0 \end{array}$	If the passenger *p* is assigned from pick-up location/drop-off location *i* to location *j*;	
	Otherwise;	
$U_{i}^{t}$	The arrival time at vertex *i* of vehicle *t*	
$x_{ij}^{pt}=\{\begin{array}{c} 1\\ 0 \end{array}$	If passenger *p* travels through arc (*i*,*j*) on-board of vehicle *t*	
	Otherwise;	
$y_{p}^{t}=\{\begin{array}{c} 1\\ 0 \end{array}$	If passenger *p* is picked up by vehicle *t*	
	Otherwise;	
$z_{ij}^{t}=\{\begin{array}{c} 1\\ 0 \end{array}$	If vehicle *t* travels through arc (*i*,*j*)	
	Otherwise;	

### 2.3 Formulation

The proposed problem is formulated into the following mixed integer linear program (MILP). To facilitate the modeling, this study considers the following assumptions:

1. Passenger walking distance, ridesharing car travel time, and travel distance are given;
2. The location of demand points and meet points are given;
3. Number of routes is given; and
4. Each ridesharing car cannot accommodate more passengers than its capacity.

#### Objective function

The objective function is expressed in the total system cost, which is the sum of the travel time of the sharing vehicle and users’ walking time to/from the pickup points.

$$Min\;Z=(\sum_{i\in V}\sum_{j\in V}\sum_{p\in P}\sum_{t\in T}x_{ij}^{pt}{Tim}_{ij})+(\sum_{t\in T}\sum_{i\in V}\sum_{p\in P}\sum_{j\in V}r_{ij}^{p}\frac{D_{ij}}{S})$$ (1)

#### Contraints

Constraints (2–5) guarantee that each passenger is assigned to one pick-up and drop off location. These constraints also prevent that vehicle routing from connecting to an unselected potential pick-up or drop-off location:

$$r_{ij}^{p}\leq d_{i},\forall i,j\in V_{p},\forall p\in P;$$ (2)

$$2*z_{ij}^{t}\leq d_{i}+d_{j},\forall t\in T,\forall i,j\in V_{p};$$ (3)

$$r_{ms}^{p}\leq d_{m},\forall m,s\in V_{d},p\in P;$$ (4)

$$2*z_{ms}^{t}\leq d_{m}+d_{s},\forall t\in T,\forall m,s\in V_{d};$$ (5)

Constraints (6–8) are used to connect passengers with rider-sharing vehicles, and ensure that each passenger is served by only one vehicle:

$$x_{ij}^{pt}\leq z_{ij}^{t},\forall t\in T,\forall i,j\in V;\forall p\in P;$$ (6)

$$\sum_{i\in V_{p}}\sum_{j\in V}x_{ij}^{pt}=1,\forall p\in P;$$ (7)

$$\sum_{m\in V}\sum_{s\in V_{d}}x_{ms}^{pt}=1,\forall p\in P;$$ (8)

Constraints (9–11) prevent any selected pick-up location being connected with multiple vehicles. The constraints further increase efficiency of the entire system:

$$\sum_{j\in V}z_{ij}^{t}\leq 1,\forall i\in V,\forall t\in T;$$ (9)

$$\sum_{j\in V}z_{ji}^{t}\leq 1,\forall i\in V,\forall t\in T;$$ (10)

$$\sum_{t\in T}y_{p}^{t}\leq 1,\forall p\in P;$$ (11)

Constraint (12) is the conservation law and ensures that each selected pick-up or drop off location has the same incoming arc and outgoing arc:

$$\sum_{i\in V}z_{jm}^{t}-\sum_{i\in V}z_{sj}^{t}\geq 0,\forall t\in T,\forall j\in V;$$ (12)

Constraints (13–14) (in the form of inequality for the ease of finding solution) guarantee the feasibility of the schedule for each vehicle trip. Specifically, it is ensured that the travel time on each arc should equal to the corresponding travel time within the given time matrix:

$$U_{j}^{t}-U_{i}^{t}\geq {Tim}_{ij}-M(1-z_{ij}^{t}),\forall i,j\in V,\forall t\in T$$ (13)

$$U_{j}^{t}-U_{i}^{t}\leq {Tim}_{ij}-M(1-z_{ij}^{t}),\forall i,j\in V,\forall t\in T$$ (14)

Where *M* is an extreme large value.

Constraint (15) guarantees the number of passengers boarded never exceeds vehicle capacity during the entire trip:

$$\sum_{p\in P}y_{p}^{t}\leq Q_{t},\forall t\in T;$$ (15)

Constraint (16) ensures that passengers are assigned to the route only if this route serves that link:

$$\sum_{p\in P}x_{ij}^{pt}\leq Q_{t}*z_{ij}^{t},\forall t\in T,\forall i,j\in V$$ (16)

Constraints (17–18) ensure that the scheduled arrival time at each pick-up point satisfies the preferred service time window of all users served at this pick-up point:

$$\max_{p\in P}\left\{e_{p}^{pick}*x_{ij}^{pt}\right)\leq U_{i}^{t}\leq \min_{p\in P}\left\{l_{p}^{pick}*x_{ij}^{pt}\right),\forall i\in V,\forall j\in V_{p},\forall t\in T;$$ (17)

$$U_{i}^{t}\leq \min_{p\in P}\left\{U_{p}^{drop}*x_{ij}^{pt}\right),\forall i\in V,\forall j\in V_{d},\forall t\in T;$$ (18)

Constraint (19) guarantees that the total travel time of vehicle *t* does not exceed the maximum travel time that driver would endure.

$$\sum_{i\in V}\sum_{j\in V}z_{ij}^{t}{Tim}_{ij}\leq T_{t},\forall t\in T;$$ (19)

Constraints (20–21) ensure that all vehicles start from ridesharing drivers’ origin locations.

$$\sum_{i\in V}\sum_{j\in V_{o}}z_{ji}^{t}=1,\forall t\in T;$$ (20)

$$\sum_{i\in V}\sum_{j\in V_{o}}z_{ij}^{t}\leq 0,\forall t\in T;$$ (21)

Constraints (22–23) guarantee that all vehicles end at ridesharing drivers’ destinations.

$$\sum_{i\in V}\sum_{j\in V_{e}}z_{ij}^{t}=1,\forall t\in T;$$ (22)

$$\sum_{i\in V}\sum_{j\in V_{e}}z_{ji}^{t}\leq 0,\forall t\in T;$$ (23)

The proposed mixed integer model is an extension of classic vehicle routing problem (VRP), which is NP-hard to resolve. The model is further complicated by the introduction of (i) meet points associated with passenger assignment and (ii) users’ preferred time windows. Therefore, it is difficult to use exact method to solve such a problem at an acceptable running time, especially for a large-scale instance. To better contend with the issue of computation efficiency, this study further proposes an enhanced Tabu Search algorithm to yield the meta-optimal solutions in a reasonable amount of time at next section.

## 3. Solution algorithm

In our case, we develop an enhanced Tabu search algorithm to solve proposed MILP model. It is based on the well-known Tabu Search (TS) algorithm, which is a memory-based search method originally developed by Glover [42]. The algorithm starts from an initial solution *s*_0_, and then moves at iteration *t* from *s*_*t*_ to the best solution in a neighborhood *N*(*s*_*t*_) of *s*_*t*_. The TS is able to avoid cycling in the search space by tracking the recent moves through the use of a memory structure called Tabu list. Moreover, to reduce the likelihood of being trapped in a local optimum, the search typically moves to the best neighboring solution even if this move allows the objective function to deteriorate results [38, 43].

### 3.1 Solution framework

The form of Tabu search we followed in this study can be tightly summarized as follows:

1. Start from a solution *s*_0_;
2. *s*_*best*_ ← *s*_0_;
3. Set the Tabu list to the empty list;
4. While a stopping criterion is not satisfied, do:
  - Generate the neighborhood of *N*(*s*_*t*_) through non-Tabu moves (or Tabu moves that lead to solutions that improve *s*_*best*_) and select the best solution *s*_*t*_;
  - If *s*_*t*_ is better than *s*_*best*_ then *s*_*best*_ ← *s*_*t*_;
  - *s*_0_ ← *s*_*t*_;
  - Update the Tabu list.
5. Output *s*_*best*_.

### 3.2 Neighborhood structure

The solutions in the neighborhood of a given solution *s* ∈ *S*, denoted as *N*(*s*), are the solutions that can be obtained by applying a single-move operation to the current solution. Each solution *s* is assigned with an attribute set *U*(*s*) = {(*p*,*t*):*passenger p is assigned to vehicle t*}. A single-move operator here is to change an attribute (*p*,*t*) from *U*(*s*) to (*p*,*t*′) where *t*′ ≠ *t*. And then, the corresponding vertices are also removed from route, in which the new vertices would be inserted. Note that, the insertion of new vertices should follow the rule of minimizing the penalized objective function value. To prevent cycling, the same passenger *p* who has been moved from vehicle *t* to vehicle *t*′ in a given iteration is forbidden to move back to vehicle *t*, or declared tabu. A tabu move is allowed only when reach a solution of smaller cost than that of the best known solution.

### 3.3 Diversification/Intensification

In our study, we follow the paper by Lai et al. [44] and integrate two diversification strategies into the Tabu search. Specifically, one is activated during the construction of the neighborhood solutions where any solution, *s* ∈ *S*, is penalized proportional to the frequency of a vertex appeared in the incumbent solution. The other is used during the search process where the penalty weights for the violation of constraints (15), (16) and (19) are dynamically adjusted for better exploration of the search space. The penalty weight *α* and *β* are initially set to 1 that are recommended by Cordeau et al. [45], and Ho and Gendreau [46]. The values of weights are then adjusted during the search according to whether the solution in the next iteration satisfies the corresponding constraints. Specifically, it is divided by (*δ* + 1) if there is no violation of the constraint; otherwise, it is multiplied by (*δ* + 1), where *δ* is a user-defined parameter.

### 3.4 Initial population

The quality of the solution found and the convergence speed of using heuristic algorithm, highly depend on the selection of the initial population. In this research, a meta-heuristic algorithm to efficiently generate a decent initial population is proposed. The procedures are explained as following:

***Step 1*.** Define parameters, such as *P* (the set of passengers), *T* (the set of vehicles) and *V*(the set of locations) etc.;

***Step 2*.** Use a backward recursion to generate a feasible routing plan for ride sharing vehicle:

1) Append tag of preferred departure time to each request by using formula:
  $$int[\frac{\left(e_{p}^{pick}+l_{p}^{pick}\right)}{2}];$$
2) Sort the requests according to the preferred arrival time, and then select the latest *T* requests, [*p*_(*P*−1)_,…,*p*_(*P*−*T*)_], with their preferred arrival and departure time;
2) For each selected request, connect its pick-up location and drop-off location, and then randomly insert another one non-selected request;
3) Repeat 2) until constraint (15), (17) or (19) is violated;

***Step 3*.** Assign passengers who are unable to be directly served to their nearest selected pick-up locations;

***Step 6*.** Generate the initial population *s*_0_ with the results from ***Step 2 to Step 3***.

### 3.5 Neighborhood evaluation

To evaluate the quality of generated route, we follow up an adapted version of the eight-step evaluation scheme introduced by [38], as recorded in Table 2. Additionally, the eight-step evaluation scheme uses the forward time slack *F*_*i*_ for a node *i* ∈ *N* defined by [47]:

$$F_{i}=\underset{i\leq j\leq q}{\min}(\sum_{i\leq p\leq j}w_{p}+{\left(\min\left(e_{p}^{pick}-U_{i}^{t},T_{t}-P_{t}\right)\right)}^{+})$$

where *w*_*p*_ denotes the waiting time at node *p*, *q* is the last node on the route, and *P*_*t*_ is the ride time of the vehicle. *F*_*i*_ gives the maximum amount of time, by which the departure from a node *i* can be delayed without violating time windows and passenger ride time.

**Table 2:** Eight-step evaluation scheme.

1. Set $U_{0}^{t}=e_{1}^{pick}$
2. Compute $U_{i(i\neq 0)}^{t}$ for each node i along vehicle route *t*
If either $U_{i(i\neq 0)}^{t}>e_{p}^{pick}$, return false
3. Compute *F*_0_ 4. Set $U_{0}^{t}=e_{1}^{pick}+\min\left(F_{0},\sum_{0\leq p\leq q}w_{p}\right)$ 5. Update $U_{i(i\neq 0)}^{t}$ 6. Compute all *P*_*t*_ If *P*_*t*_ ≤ *T*_*t*_ return true 7. Compute departure time for nodes where are origins 8. Return false

### 3.6 Stopping criterion

In our case, two stopping criteria have been applied, i.e., the run time limit and the maximum number of consecutive iterations (2,000).

## 4. Numerical experiments

The aim of this section is to verify the applicability of the proposed model and the efficiency of the developed meta-heuristic algorithm. In the following, the detailed information about the experimental design (e.g., network layout and operation scenarios) is first introduced and then followed by the numerical results.

### 4.1 Experimental design

#### 4.1.1 Network layout

**Fig 1:**
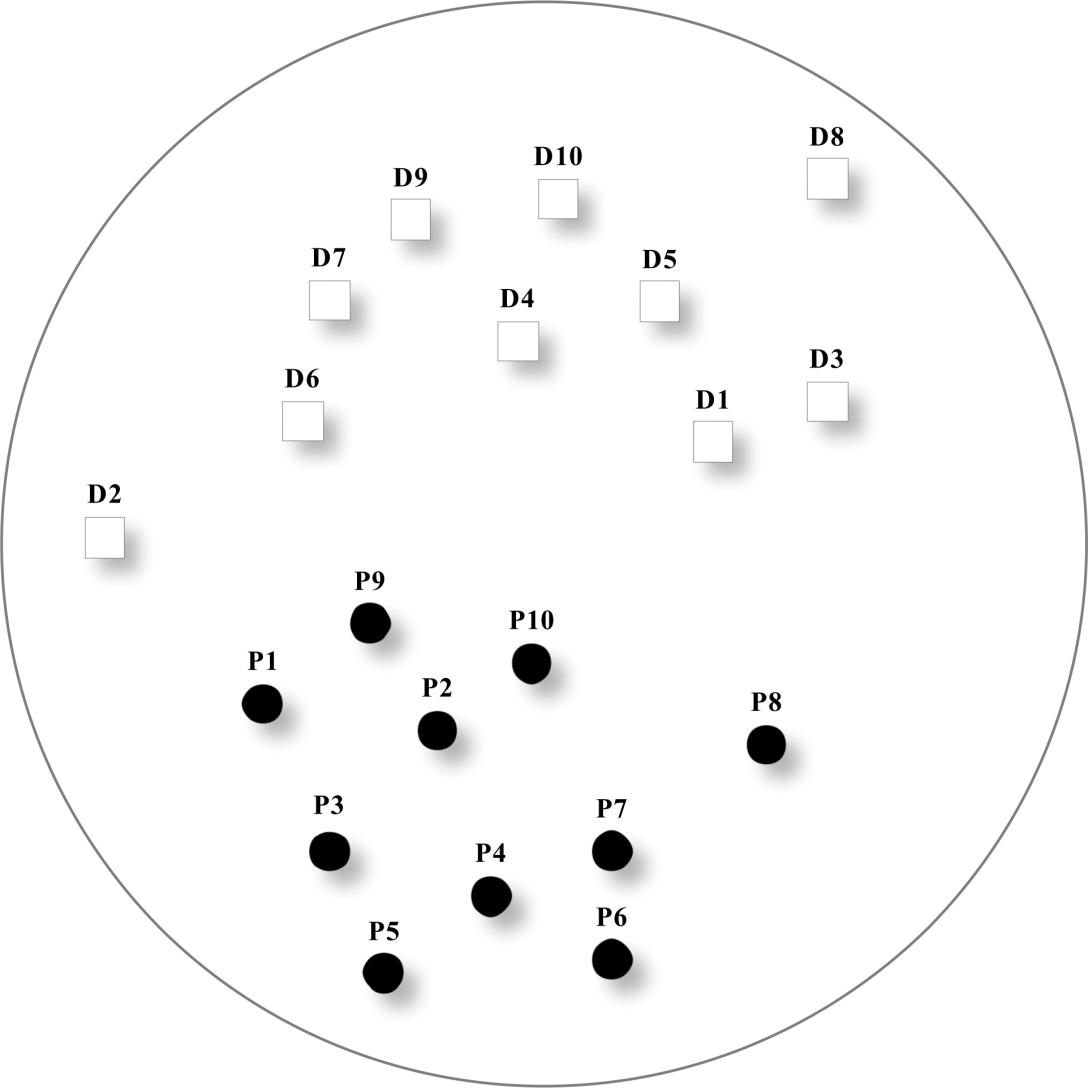
Spatial distribution of pick-up and drop-off locations.

The proposed ridesharing system is tested on a manipulated network (as shown in Fig 1), in which 10 ride requests are appended to10 pick-up locations (shown by black solid circles), and another 10 drop-off locations (hollow squares) are also designed. Each ride request is assigned with the preferred service time window of pick-up and drop-off, as well as one drop-off location, as given in Table 3.

**Table 3:** Ride request information.

Ride request locations	Preferred boarding time window	Desired drop-off location	Preferred arrival time
P1	5:55–6:10	D1	6:20
P2	5:55–6:10	D2	6:20
P3	6:00–6:05	D3	6:15
P4	6:00–6:10	D4	6:20
P5	5:55–6:05	D5	6:15
P6	6:00–6:05	D6	6:25
P7	6:00–6:05	D7	6:20
P8	6:05–6:10	D8	6:15
P9	6:00–6:10	D9	6:20
P10	6:05–6:10	D10	6:20

#### 4.1.2 Scenario design

A total of three scenarios are designed to show the advantage of the proposed model and the Tabu-based meta-heuristic solver. The designed three scenarios will test the service performance of the ridesharing system when the fleet has 3 ridesharing cars, 4 ridesharing cars, and 5 ridesharing cars, respectively. In all scenarios, the proposed MILP model is solved by both the out-of-the-shelf commercial solver CPLEX 12.6 and by the proposed Tabu-based meta-heuristic solver. The program is run on a computer with Windows 8, a 2.8 GHz processor, and an 8 GB of RAM. The results comparison will be further unfolded to show the efficiency of the proposed meta-heuristic algorithm.

To simplify the experiments, the origins and destinations of ridesharing car drivers are randomly selected from the sets of pick-up and drop-off locations. Note that comparing with the scenario of 3-car, the 4-car and 5-car scenarios add new drivers while the information of the existing drivers remains the unchanged (as shown in Table 4).

**Table 4:** Ridesharing driver information.

Scenario	Driver	Origin	Destitution	Preferred departure time	Preferred arrival time
3-car	1	P1	D1	6:00–6:10	6:25
	2	P2	D6	5:55–6:15	6:30
	3	P3	D8	5:50–6:05	6:25
4-car	1	P1	D1	6:00–6:10	6:25
	2	P2	D6	5:55–6:15	6:30
	3	P3	D8	5:50–6:05	6:25
	4	P4	D7	5:55–6:05	6:20
5-car	1	P1	D1	6:00–6:10	6:25
	2	P2	D6	5:55–6:15	6:30
	3	P3	D8	5:50–6:05	6:25
	4	P4	D7	5:55–6:05	6:20
	5	P5	D4	6:00–6:10	6:30

Other key parameters used in the case study include: the vehicle capacity is set to be 4 persons per vehicle; the maximum allowed travel time is 40 mins per route; and the average walking speed is 5 km/h.

Note that in practice to mitigate impact of travel time uncertainties, the input travel time matrix can be generated from an average travel time of the past three days with using commercial map engine (e.g., Baidu Map). Upon the future research interest, the proposed model is able to be embedded with map engine that reads the real time travel time information, so as to further alleviate the side effects of travel time uncertainty.

### 4.2 Experiments results

#### 4.2.1 Comparisons between CPLEX and meta-heuristic results

We run CPLEX and the proposed Tabu-based meta-heuristic algorithm to solve the models under the three scenarios, respectively. The results are summarized in Tables 5–7, in which the meta-heuristic solutions are used to illustrate the effectiveness of the proposed algorithm by comparing with the global optima obtained by CPLEX.

**Table 5:** Solution of 3-car scenario.

Vehicle	Driver origin/ Departure time	Pick-up / Service time				Drop-off / Arrival time			Driver destination/ Arrival time	Passenger assignment/ Walking time (mins)	Travel time (mins)
CPLEX Solution (obtained in 750 CPU seconds)											
1^st^	P1	P6	P7	P4		D6	D7	D4	D1	-	21
	5:58	6:02	6:04	6:06		6:11	6:14	6:16	6:19	-	
2^nd^	P2	P2	P9	P10	P8	D8	D10	D9	D6	-	24
	6:00	6:00	6:01	6:04	6:06	6:11	6:14	6:16	6:24	-	
3^rd^	P3	P3	P1			D3	D5	D1	D8	P5→P3	28
	6:02	6:02	6:05			6:11	6:13	6:15	6:20	10	
Total travel time (mins)											73
Meta-heuristic Solution (obtained in 102 CPU seconds)											
1^st^	P1	P9	P10	P4	P8	D8	D10	D4	D1	D9→D4	31
	6:00	6:03	6:05	6:07	6:10	6:15	6:18	6:20	6:23	8	
2^nd^	P2	P2	P6	P7		D6	D7	D2	D6	-	19
	6:00	6:03	6:05	6:07		6:10	6:13	6:15	6:19	-	
3^rd^	P3	P3	P1			D3	D5	D1	D8	P5→P3	29
	6:01	6:01	6:04			6:10	6:12	6:14	6:19	10	
Total travel time (mins)											79
Difference with the CPLEX result (%)											8%

**Table 6:** Solution of 4-car scenario.

Vehicle	Driver origin/ Departure time	Pick-up / Service time			Drop-off / Arrival time			Driver destination/ Arrival time	Passenger assignment/ Walking time (mins)	Total Time (mins)
CPLEX Solution (obtained in 1,566 CPU seconds)										
1^st^	P1	P9	P10		D9	D10		D1	-	14
	6:02	6:04	6:06		6:10	6:12		6:16	-	
2^nd^	P2	P2			D2			D6	-	13
	6:04	6:06			6:10			6:17	-	
3^rd^	P3	P3	P5	P1	D4	D5	D1	D8	P4→P5; D3→D1	30
	6:01	6:01	6:03	6:06	6:10	6:12	6:14	6:19	12	
4^th^	P4	P6	P7	P8	D8	D7	D6	D7	-	20
	5:58	6:02	6:04	6:05	6:10	6:14	6:15	6:18	-	
Total travel time (mins)										77
Meta-heuristic Solution (obtained in 159 CPU seconds)										
1^st^	P1	P2	P9	P10	D10	D9	D2	D1	-	26
	5:55	6:00	6:02	6:05	6:07	6:09	6:13	6:21	-	
2^nd^	P2	P4	P8		D8	D4		D6	-	20
	6:04	6:07	6:10		6:15	6:19		6:24	-	
3^rd^	P3	P3	P1		D3	D1		D8	-	18
	6:02	6:02	6:05		6:12	6:15		6:20	-	
4^th^	P4	P5	P6	P7	D5	D6	D7	D7	-	21
	5:57	6:00	6:02	6:04	6:11	6:15	6:18	6:18	-	
Total travel time (mins)										85
Difference with the CPLEX result (%)										10%

**Table 7:** Solution of 5-car Scenario.

Vehicle	Driver origin/ Departure time	Pick-up / Service time			Drop-off / Arrival time			Driver destination/ Arrival time	Passenger assignment/ Walking time (mins)	Total time (mins)
CPLEX Solution (obtained in 4210 CPU seconds)										
1^st^	P1	P1	P3	P5	D1	D3	D5	D1	-	18
	6:00	6:00	6:03	6:05	6:11	6:13	6:15	6:18	-	
2^nd^	P2	P2			D2			D6	-	8
	6:10	6:10			6:14			6:18	-	
3^rd^	P3	P9	P10		D10	D9		D8	-	19
	6:01	6:04	6:06		6:10	6:12		6:20	-	
4^th^	P4	P4	P8		D8	D4		D7	-	15
	6:02	6:02	6:05		6:10	6:13		6:17	-	
5^th^	P5	P6	P7		D6	D7		D4	-	15
	6:02	6:04	6:05		6:10	6:13		6:17	-	
Total travel time (mins)										75
Meta-heuristic Solution (obtained in 266 CPU seconds)										
1^st^	P1	P1	P3		D3	D1		D1	-	13
	6:02	6:02	6:05		6:12	6:15		6:15	-	
2^nd^	P2	P2	P10		D10	D2		D6	-	18
	6:00	6:00	6:02		6:07	6:14		6:18	-	
3^rd^	P3	P4	P8		D8	D4		D8	-	23
	6:03	6:07	6:10			6:15	6:19		6:26		-
4^th^	P4	P6	P7		D6	D7		D7	-	15
	5:58	6:02	6:04		6:10	6:13		6:13	-	
5^th^	P5	P5	P9		D5	D9		D4	-	17
	6:00	6:00	6:04		6:10	6:14		6:17	-	
Total travel time (mins)										86
Difference with the CPLEX result (%)										15%

Table 5 gives the solutions of the 3-car scenario by the two solvers. Taking the route of the first vehicle in CPLEX solution as an instance, the driver departs from his/her origin, P1, at 5:58, then picks users at P6 (6:02), P7 (6:04), and P4 (6:06) in sequence, then transport them to their desired drop-off location, D6 (6:11), D7 (6:14), and D4 (6:16), respectively. Finally the first driver terminates the service at his/her own destination, D1, at 6:19. In CPLEX solution, one passenger assignment is observed in the route of vehicle 3, where the user at P5 is suggested to walk to P3 (which takes 10 mins) for the ridesharing service. This assignment benefits in reducing the total system cost. The optimal objective value of 3-car scenario, including in-vehicle time and walking time, is 73 mins. Comparatively, the solution of the proposed algorithm suggests another passenger assignment: the user at P9, whose desired drop-off location is D9, is suggested to alight at D4 (along with users from P4) and then walk to his/her own destination (i.e., D9). The objective value yields 79 mins.

Table 6 presents the solutions of the 4-car ridesharing system by two solvers, respectively. Two passenger assignments are witnessed in the CPLEX solution (i.e., in route of vehicle 3, P4 user is assigned to P5 pick-up location, and P3 users to alight at D1), whereas the meta-heuristic solution suggests no passenger assignment, and thus results in more en-route detour and longer vehicular distance in the ride sharing service (see the total travel time of 85 mins that is higher than that of CPLEX result).

Table 7 reveals the model results of the 5-car ridesharing system. Both of the CPLEX and meta-heuristic solutions suggest that there is no need to assign any passengers walking to other pick-up or drop-off locations when there are 5 cars in service. The difference between the CPLEX solution and our meta-heuristic solution is 11mins (75 mins vs. 86 mins).

From the above results, it is demonstrated that the proposed algorithm is able to provide reasonably good solutions (with in 8%~15% difference from the global optima) under all scenarios. Consistent conclusion is obtained by both the CPLEX and meta-heuristic solutions: The most efficient ridesharing system is the 3-car operation with the minimum objective value (i.e., 73 mins) in serving 10 requests. Thus, the 4-car and 5-car ridesharing systems appear to have redundant fleet assigned in the service. Additionally, the proposed algorithm shows much more advantage in its computation efficiency, which does not increase (from 102 s to 266 s) so rapidly as the CPLEX solver (from 750 s to 4,210 s). The proposed algorithm stably generates the near optimal solutions within 5 minutes, implying the insensitivity to the network complexity.

#### 4.2.2 Comparison between “with” and “without” the setting of meet points

To illustrate the merits of introducing meet points into the ridesharing system, we compare scenarios “with” and “without” meet points. For the scenario without meet points, the following constraint is added into the proposed model:

$$\sum_{{i\in V}_{p}}\sum_{j\in V_{p}}\sum_{p\in P}r_{ij}^{p}=\sum_{i{\in V}_{p}}d_{i}$$ (24)

Constraint (24) restricts that a passenger can only be assigned to one pickup/drop-off point (i.e., his/her own origin and destination). We conduct the comparisons for 3-car and 4-car ridesharing systems with the global optimal solutions obtained by CPLEX solver. The results are presented in Tables 8 and 9, respectively. It is seen that every passenger is picked up and dropped off at his/her own origin and destination under the scenario without meet points. The optimal routing plans differ from that of the scenario with meeting points. Comparatively, excluding meet points increases the total travel time by 2.7% and 3.8% for the 3-car and 4-car ridesharing systems, respectively. More considerable merits of introducing meet points are expected for ridesharing systems with large size of fleet under day-to-day operation.

**Table 8:** Comparison of 3-car scenario with and without meet points.

Vehicle	Driver origin/ Departure time	Pick-up / Service time				Drop-off / Arrival time				Driver destination/ Arrival time	Passenger assignment/	Travel time
											Walking time (mins)	(mins)
CPLEX Solution (obtained in 750 CPU seconds) "with" meet points												
1^st^	P1	P6	P7	P4		D6	D7	D4		D1	-	21
	05:58	06:02	06:04	06:06		06:11	06:14	06:16		06:19	-	
2^nd^	P2	P2	P9	P10	P8	D8	D10	D9		D6	-	24
	06:00	06:00	06:01	06:04	06:06	06:11	06:14	06:16		06:24	-	
3^rd^	P3	P3	P1			D3	D5	D1		D8	P5→P3	28
	06:02	06:02	06:05			06:11	06:13	06:15		06:20	10	
Total travel time (mins)												73
CPLEX Solution (obtained in 870 CPU seconds) "without" meet points												
1^st^	P1	P10	P4	P8		D8	D10	D4		D1	-	28
	05:55	06:05	06:07	06:10		06:15	06:18	06:20		06:23	-	
2^nd^	P2	P2	P6	P7		D6	D7	D2		D6	-	24
	05:58	05:58	06:05	06:07		06:10	06:13	06:15		06:22	-	
3^rd^	P3	P3	P5	P1	P9	D3	D5	D1	D9	D8	-	23
	06:01	06:01	06:03	06:06	06:09	06:12	06:14	06:17	06:19	06:24	-	
Total travel time (mins)												75
Difference with the CPLEX result (%)												2.7%

**Table 9:** Comparison of 4-car scenario with and without meet points.

Vehicle	Driver origin/ Departure time	Pick-up / Service time			Drop-off / Arrival time			Driver destination/ Arrival time	Passenger assignment/ Walking time (mins)	Total Time (mins)
CPLEX Solution (obtained in 1,566 CPU seconds) with "meet points"										
1^st^	P1	P9	P10		D9	D10		D1	-	14
	06:02	06:04	06:06		06:10	06:12		06:16	-	
2^nd^	P2	P2			D2			D6	-	13
	06:04	06:06			06:10			06:17	-	
3^rd^	P3	P3	P5	P1	D4	D5	D1	D8	P4→P5; D3→D1	30
	06:01	06:01	06:03	06:06	06:10	06:12	06:14	06:19	12	
4^th^	P4	P6	P7	P8	D8	D7	D6	D7	-	20
	05:58	06:02	06:04	06:05	06:10	06:14	06:15	06:18	-	
Total travel time (mins)										77
CPLEX Solution (obtained in 2601 CPU seconds) without "points"										
1^st^	P1	P9	P10		D9	D10		D1	-	14
	06:02	06:04	06:06		06:10	06:12		06:16	-	
2^nd^	P2	P2	P4		D2	D4		D6	-	22
	06:04	06:06	06:10		06:16	06:19		06:26	-	
3^rd^	P3	P3	P5	P1	D5	D3	D1	D8	-	24
	06:01	06:01	06:03	06:06	06:12	06:15	06:18	06:25	-	
4^th^	P4	P6	P7	P8	D8	D7	D6	D7	-	20
	05:58	06:02	06:04	06:05	06:10	06:14	06:15	06:18	-	
Total travel time (mins)										80
Difference with the CPLEX result (%)										3.8%

## 5. Conclusions

The paper presents a mixed integer linear programming model for the route design of an enhanced ridesharing system with meet points for collective pickups and drop-offs. In the modeling, practical constraints, e.g., users’ preferable time windows and drivers’ maximum acceptable travel time, are respected. The current problem is an extension of the traditional dial-a-ride problem (DARP), for which passenger assignment model has been developed in trade-off the additional walking time and the route travel time saving (due to reduced number of stops). An enhanced Tabu-based meta-heuristic algorithm is developed to solve the problem within an acceptable running time. The algorithm integrates two diversification strategies and eight-step evaluation framework to enhance the searching ability. Moreover, an additional heuristic algorithm is also designed and embedded into Tabu search to generate the feasible initial population. To evaluate the effectiveness of the proposed algorithm, the model is also solved to optimality using CPLEX. Three scenarios (i.e., 3-car, 4-car, and 5-car ridesharing systems) are designed to conduct the numerical experiments in a complete network with 20 vertices (10 are pickup locations and 10 drop-off locations). The results demonstrate that the model can provide detailed route plan for ridesharing drivers as well as meet point arrangement for users. Compared with the solutions obtained by CPLEX, the proposed algorithm is shown to be able to yield high-quality solutions (with difference in the range of 8%-15%) for all scenarios. In terms of the computation efficiency, the meta-heuristic algorithm shows a remarkable advantage over the CPLEX. In practice, given the constraints in time and level of service, ridesharing operators can easily make trade-off between the solution quality and the computational time by placing the penalty to neighborhood evaluation in the proposed algorithm.

Future research will be explored in the following directions: (i) mining the travel pattern using mobile-source data [48] to better identify the meet points and develop sophisticated passenger assignment plan, (ii) embedding traffic information (e.g., speed) [49–52] into the route planning of the ridesharing system to furnish improved service in congested urban area; and (iii) integrating the ridesharing system as feeder service to conventional public transit with emphasis on the schedule synchronization problem [53–55].

## References

[1] Desaulniers G., Desrosiers J., Erdmann A., Solomon MM., Soumis F. The VRP with pickup and delivery In: Toth P., Vigo D., editors. The Vehicle Routing Problem, SIAM Monographs on Discrete Mathematics and Applications, Chapter 9. SIAM, Philadelphia; 2002 pp. 225–242.

[2] Mahmoudi M., Zhou X. Finding optimal solutions for vehicle routing problem with pickup and delivery services with time windows: A dynamic programming approach based on state–space–time network representations. Transportation Research Part B: Methodological. 2016; 89:19–42.

[3] Zachariadis E., Tarantilis C., Kiranoudis C. The load-dependent vehicle routing problem and its pick-up and delivery extension. Transportation Research Part B: Methodological. 2015; 71:158–181.

[4] Solomon M.M., Chalifour A., Desrosiers J., Boisvert J. An application of vehicle routing methodology to large-scale larvicide control programs. Interfaces. 1992; 22: 88–99.

[5] Rappoport H.K., Levy L.S., Golden B.L., Toussaint K. A planning heuristic for military airlift. Interfaces. 1992: 22, 73–87.

[6] Rappoport H.K., Levy L.S., Toussaint K., Golden B.L. A transportation problem formulation for the MAC airlift planning problem. Annals of Operations Research. 1994; 50: 505–523.

[7] Ioachim I., Desrosiers J., Dumas Y., Solomon M.M., Villeneuve D. A request clustering algorithm for door-to-door handicapped transportation. Transportation Science. 1995; 29: 63–78.

[8] Toth P., Vigo D. Heuristic algorithms for the handicapped persons transportation problem. Transportation Science. 1997; 31: 60–71.

[9] Bramel J., Simchi-Levi D. A location based heuristic for general routing problems. Operations Research. 1995; 43: 649–660.

[10] Swersey A., Ballard W. Scheduling school buses. Management Science. 1983; 30: 844–853.

[11] Agatz N., Erera A., Savelsbergh M., Wang X. Optimization for dynamic ridesharing: A review. European Journal of Operational Research. 2012; 223:295–303.

[12] Psaraftis HN. A dynamic programming solution to the single vehicle many-to-many immediate request dial-a-ride problem. Transportation Science. 1980; 14:130–154.

[13] Psaraftis HN. An exact algorithm for the single vehicle many-to-many dial-a-ride problem with time windows. Transportation Science. 1983; 17: 351–357.

[14] Desrosiers J., Dumas Y., Soumis F. A dynamic programming solution of the large- scale single-vehicle dial-a-ride problem with time windows. American Journal of Mathematical and Management Sciences. 1986; 6:301–325.

[15] Ziliaskopoulos, A., Kozanidis, G. Dynamic programming strategies for the dial a ride problem with time window constraints. In: 85th Annual Meeting of the Transportation Research. 2006; p.1-12.

[16] Lois, A., Ziliaskopoulos, A., Aifantopoulou, G. A very large scale neighborhood heuristic algorithm for the multivehicle dial a ride with time windows. In: Proceedings of 87th Transportation Research Board Annual Meeting. 2007; p.1-16.

[17] Cordeau JF., Laporte G. The dial-a-ride problem (DARP): Variants, modeling issues and algorithms. 4OR: A Quarterly Journal of Operations Research. 2003; 1:89–101.

[18] Masmoudi M.A., Braekers K., Masmoudi M., Dammak A. A Hybrid Genetic Algorithm for the Heterogeneous Dial-A-Ride Problem. Computers & operations research. 2017; 81:1–13.

[19] Masson R., Lehuede F., Peton O. The dial-a-ride problem with transfers. Computers & Operations Research. 2014; 41:12–23.

[20] Malandraki C., Daskin MS. Time Dependent Vehicle Routing Problems: Formulations, Properties and Heuristic Algorithms. Transportation Science. 1992; 26: 185–199.

[21] Fu L. Scheduling dial-a-ride paratransit under time-varying, stochastic congestion. Transportation Research Part B. 2002; 36:485–506.

[22] Molenbruch Y., Braekers K., Caris A. Typology and literature review for dial- a-ride problems. Annals of Operations Research. 2017; p.1–31.

[23] Diana M., Dessouky MM., Xia N. A model for the fleet sizing of demand responsive transportation services with time windows. Transportation Research Part B: Methodological. 2006; 40:651–666.

[24] Attanasio A., Cordeau JF., Ghiani G., Laporte G. Parallel tabu search heuristics for the dynamic multi-vehicle dial-a-ride problem. Parallel Computing. 2004; 30:377–387.

[25] Berbeglia G., Cordeau J.-F., Laporte G. A hybrid tabu search and constraint programming algorithm for the dynamic dial-a-ride problem. INFORMS Journal on Computing. 2012; 24:343–355.

[26] Feuerstein E., Stougie L. On-line single-server dial-a-ride problems. Theoretical Com- puter Science. 2001; 268: 91–105.

[27] Diana M., Dessouky MM. A new regret insertion heuristic for solving large-scale dial- a-ride problems with time windows. Transportation Research Part B: Methodological. 2004; 38:539–557.

[28] Dumas Y., Desrosiers J., Soumis F. The pickup and delivery problem with time windows. European Journal of Operational Research. 1991; 54:7–22.

[29] Cordeau JF. A Branch-and-cut algorithm for the dial-a-ride problem. Operations Research. 2006; 54:573–586.

[30] Ropke S., Cordeau JF. Branch and Cut and Price for the Pickup and Delivery Problem with Time Windows. Transportation Science. 2009; 43:267–286.

[31] Jaw JJ., Odoni AR., Psaraftis HN., Wilson NHM. A heuristic algorithm for the multi-vehicle advance-request dial-a-ride problem with time windows. Transportation Research Part B: Methodological. 1986; 20:243–257.

[32] KimT, Haghani A. Model and Algorithm Considering Time-Varying Travel Times to Solve Static Multi depot Dial- a-Ride Problem. In: Proceedings of 87th Transportation Research Board Annual Meeting. 2011; p.68-77.

[33] Wong KI., Han AF., Yuen CW. On dynamic demand responsive transport services with degree of dynamism. Transportmetrica A-Transport Science. 2014; 10: 55–73.

[34] Luo Y., Schonfeld P. Online rejected-reinsertion heuristics for dynamic multivehicle dial-a-ride problem. Transportation Research Record: Journal of the Transportation Research Board. 2011; p.59–67.

[35] Ioachim I., Desrosiers J., Dumas Y., Solomon MM., Villeneuve D. A request clustering algorithm for door-to-door handicapped transportation. Transportation Science. 1995; 29:63–78.

[36] Borndorfer R., Grotschel M., Klostermeier F., Kuttner C. Telebus Berlin: Vehicle scheduling in a dial-a-ride system. Lecture Notes in Economics and Mathematical Systems. 1999; 471:391–422.

[37] Karabuk S. A nested decomposition approach for solving the paratransit vehicle scheduling problem. Transportation Research Part B: Methodological. 2009; 43:448–465.

[38] Cordeau JF., Laporte G. A tabu search heuristic for the static multi-vehicle dial-a- ride problem. Transportation Research Part B: Methodological. 2003; 37:579–594.

[39] Stiglic M., Agatz N., Savelsbergh M., Gradisar M. The benefits of meeting points in ridesharing systems. Transportation Research Part B: Methodological. 2015; 82: 36–53.

[40] Daganzo CF. Checkpoint dial-a-ride systems. Transportation Research Part B: Methodological. 1984; 18:315–327.

[41] Zhao J., Liu Y., Li P. A network enhancement model with integrated lane reorganization and traffic control strategies. Journal of Advanced Transportation. 2016; 50(6): 1090–1110.

[42] Glover F. Future paths for integer programming and links to artificial intelligence. Computers & operations research. 1986; 13: 533–549.

[43] Kirchler D., Calvo RW. A granular tabu search algorithm for the dial-a-ride problem. Transportation Research Part B: Methodological. 2013; 56:120–135.

[44] Lai DS., Demirag OC., Leung JM. A tabu search heuristic for the heterogeneous vehicle routing problem on a multigraph. Transportation Research Part E: Logistics and Transportation Review. 2016; 86:32–52.

[45] Cordeau JF., Laporte G., Mercier A. A unified tabu search heuristic for vehicle routing problems with time windows. Journal of the Operational research society. 2001; 52:928–936.

[46] Ho SC., Gendreau M. Path relinking for the vehicle routing problem. Journal of Heuristics, 2006; 12: 55–72.

[47] Savelsbergh MW. Local search in routing problems with time windows. Annals of Operations research. 1985; 4: 285–305.

[48] Ma X., Wu Y., Wang Y., Chen F., Liu J. Mining smart card data for transit riders' travel patterns. Transportation Research Part C: Emerging Technologies. 2013; 36:1–12

[49] Abdel-Aty MA., Kitamura R., Jovanis PP. Using stated preference data for studying the effect of advanced traffic information on drivers' route choice. Transportation Research Part C: Emerging Technologies. 1997; 5:39–50.

[50] Fleischmann B., Gnutzmann S., Sandvoß E. Dynamic vehicle routing based on online traffic information. Transportation science. 2004; 38:420–433.

[51] Nzouonta J., Rajgure N., Wang G., Borcea C. VANET routing on city roads using real-time vehicular traffic information. IEEE Transactions on Vehicular technology. 2009; 58: 3609–3626.

[52] Ma X., Dai Z., He Z., Ma J., Wang Y., Wang Y. Learning traffic as images: A deep convolution neural network for large-scale transportation network speed prediction. Sensors. 2017; 17:818. 10.3390/s17040818 PMC5422179 28394270

[53] Brake J., Mulley C., Nelson JD., Wright S. Key lessons learned from recent experience with flexible transport services. Transport Policy. 2007; 14:458–466.

[54] Tangphaisankun, A., Nakamura, F., Okamura, T. Influences of paratransit as a feeder of mass transit system in developing countries based on commuter satisfaction. In: Proceedings of the Eastern Asia Society for Transportation Studies Vol. 7 (The 8th International Conference of Eastern Asia Society for Transportation Studies.) 2009; p. 236–236.

[55] Ma X., Chen X., Ding C., Wang Y. Sustainable station-level planning: an integrated transport and land use design model for transit-oriented development. Journal of Cleaner Production. 2018; 170: 1052–1063.

